# Rescuing mesenchymal stem cell regenerative properties on hydrogel substrates post serial expansion

**DOI:** 10.1002/btm2.10104

**Published:** 2018-09-20

**Authors:** Varsha V. Rao, Michael K. Vu, Hao Ma, Anouk R. Killaars, Kristi S. Anseth

**Affiliations:** ^1^ Dept. of Chemical and Biological Engineering University of Colorado Boulder CO, 80303; ^2^ BioFrontiers Institute University of Colorado Boulder CO, 80303; ^3^ Dept. of Materials Science and Engineering University of Colorado Boulder CO, 80309

**Keywords:** cell manufacturing, hydrogels, mechanotransduction, mesenchymal stem cells

## Abstract

The use of human mesenchymal stem/stromal cells (hMSCs) in most clinical trials requires millions of cells/kg, necessitating *ex vivo* expansion typically on stiff substrates (tissue culture polystyrene [TCPS]), which induces osteogenesis and replicative senescence. Here, we quantified how serial expansion on TCPS influences proliferation, expression of hMSC‐specific surface markers, mechanosensing, and secretome. Results show decreased proliferation and surface marker expression after five passages (P5) and decreased mechanosensing ability and cytokine production at later passages (P11‐P12). Next, we investigated the capacity of poly(ethylene glycol) hydrogel matrices (E ~ 1 kPa) to rescue hMSC regenerative properties. Hydrogels reversed the reduction in cell surface marker expression observed at P5 on TCPS and increased secretion of cytokines for P11 hMSCs. Collectively, these results show that TCPS expansion significantly changes functional properties of hMSCs. However, some changes can be rescued by using hydrogels, suggesting that tailoring material properties could improve *in vitro* expansion methods.

## 1 INTRODUCTION

Human mesenchymal stem/stromal cells (hMSCs) are multipotent cells capable of differentiating into cell types found in tissues of the mesoderm (bone, cartilage, and fat), ectoderm (epithelium and neural), and endoderm (muscle, gut, and lung).^1^ hMSCs are characterized by a cell surface maker profile, which is constituted by the positive expression of CD105, CD90, and CD73 and negative expression of CD34, CD45, and CD14.^1^ hMSCs are also capable of secreting various cytokines and chemokines to modulate immune responses and promote wound healing. As a result of their myriad capabilities in regenerative therapies, hMSCs are one of the most widely used stem cells in clinical trials with over 800 trials registered worldwide.^2^ hMSCs are being tested as cell‐based therapies for the treatment of graft versus host disease, myocardial infraction, various neurological diseases, and bone and cartilage regeneration. Although the number of trials using hMSCs has increased three‐fold over the past decade, the percentage of these trials that have advanced to Phase III/IV has stagnated around 2%–7% for multiple years.^3^, ^4^ While the lack of late phase trials is the result of many compounding problems, one contributing factor is a lack of robust, scalable, and reproducible methods that allow efficient *ex vivo* expansion of hMSCs while maintaining their therapeutic properties.^4^, ^5^, ^6^, ^7^ hMSCs readily adhere to tissue culture plastic surfaces, a property used in their isolation from bone marrow where they make up approximately 0.001%–0.01% of mononuclear cells. Isolation alone does not yield enough cells for fundamental studies and/or clinical applications. Thus, hMSCs are expanded *ex vivo*. This is typically done on stiff surfaces like tissue culture polystyrene (TCPS) in laboratory settings. Multilayered TCPS flasks or polystyrene microbeads in bioreactors are often used for clinical scale expansion. A typical intravenous dose of hMSCs is approximately 1 million cells/kg for each patient. Thus, the clinical use of hMSCs is contingent upon their successful *ex vivo* expansion.^8^, ^9^

While regenerative medicine applications exploit the multipotency and differentiation of hMSCs, they are also known to secrete many trophic factors that impact therapeutic outcomes. hMSCs secrete various cytokines and chemokines involved in immunodulation, especially those related to inflammation signaling, cell trafficking, and lymphocyte differentiation and proliferation.^10^, ^11^, ^12^, ^13^ Although the precise mechanisms involved in hMSC immunomodulation are largely unknown, several molecules, such as TNF‐α and IL‐6,^14^, ^15^ have been cited as potent regulators of initial inflammatory responses, while others, such as VEGF or HGF,^10^, ^16^, ^17^ can aid in angiogenesis and wound healing. Additionally, hMSCs can suppress immune responses and promote tissue homeostasis by secreting PGE2 or IDO to promote M2 macrophage polarization.^15^

hMSCs have the ability to sense the mechanics of their environment through integrins that translate extracellular mechanical cues into intracellular biochemical signaling. One output of this mechanotransduction is the nuclear shuttling of yes‐associated protein (YAP) on culture substrates with high elastic moduli.^18^ Many studies have used biomaterials with tunable elastic moduli and viscoelasticity to investigate the influence of these mechanical properties on the differentiation of hMSCs. For example, hMSCs have been shown to commit to a single cell lineage when cultured on substrates with moduli corresponding to tissue‐specific matrix properties (e.g., *E* ~ 0.1 kPa for neurogenesis, *E* ~ 10 kPa for myogenesis, and *E* > 25 kPa for osteogenesis).^19^ Following up on this work, Yang et al.^20^ found that mechanosensing ability of hMSCs, and ultimately their multipotency, depended on the time of exposure to stiff matrix environments. Uninterrupted culture on substrates with stiff moduli (*E* ~ 40 kPa) for 10 days caused irreversible YAP nuclear localization, even when the substrate was in situ softened (*E* ~ 2 kPa). However, the effects were reversible when the exposure to the stiff microenvironment was shorter (<7 days).^20^ Additionally, cells exposed to longer stiff mechanical doses were biased towards osteogenesis, losing their multipotency. The time course of matrix stiffness has also been shown to influence angiogenesis.^17^ hMSCs cultured on 4 kPa hydrogels showed increased mRNA levels of genes associated with new blood vessel formation compared to mRNA levels of hMSCs cultured on hydrogels of lower modulus.^21^ hMSCs primed on soft hydrogels (~2 to 5 kPa) show reduced α‐smooth muscle actin expression, a marker for a pro‐fibrotic response, even after transfer to stiff hydrogels (100 kPa), indicating soft mechanical memory.^22^ Previous studies have indicated that prolonged culture on traditional tissue culture plates and the use of enzymatic passaging methods can bias hMSCs toward an osteogenic fate,^23^ cause loss of chondrogenic and adipogenic differentiation ability,^23^, ^24^ cause loss of DNA repair ability,^25^ induce replicative senescence, and decrease cell surface markers essential to hMSC function.^23^, ^26^, ^27^ Less is known about the effect that prolonged expansion on stiff surfaces may have on hMSC mechanosensing and secretory properties. Motivated by the growing body of evidence that hMSCs respond to both the magnitude and dose of their substrate modulus, we sought to further characterize the temporal changes that occur in hMSC properties both under typical expansion conditions and when transferred to hydrogel substrates.

When hMSCs are isolated from their bone marrow niche and expanded *ex vivo*, stimuli from the culture microenvironment can intentionally or unintentionally influence their regenerative properties, ultimately affecting the potency of transplanted hMSCs. This motivated the fundamental studies reported herein, where we sought to characterize and quantify phenotypic drift in hMSCs during their expansion under typical conditions used in research laboratories. The characterization focused on defined *in vitro* criteria based on properties of freshly isolated hMSCs: proliferation, cell surface marker expression, mechanosensing abilities, and secretome. The drift of each of these properties was quantified during expansion on TCPS with repeated enzymatic detachment. Uniquely, the experimental design included conditions to investigate the effect of soft poly(ethylene glycol) (PEG)‐hydrogel usage in the expansion protocol on the hMSC phenotype. hMSCs of early (P1–P2), middle (P5–P7), and late (P11–P12) passages were transferred to PEG‐hydrogels (*E* ~ 1 kPa) post TCPS expansion. Together, we hypothesized that exposure to soft matrix cues after serial passaging on TCPS could recover or maintain the regenerative and multipotency properties of hMSCs lost during expansion.

## 2 MATERIALS AND METHODS

### 2.1 hMSC isolation and expansion

Fresh human bone marrow aspirate was purchased from Lonza (donor 18‐year‐old black female) and the hMSCs were isolated based on preferential adhesion to TCPS plates, using previous published protocols.^20^, ^28^ Freshly isolated hMSCs (P0) were detached with 0.05% trypsin–EDTA (Sigma) and subsequently centrifuged, counted, and frozen down in 80% fetal bovine serum (FBS; Invitrogen) and 20% dimethylsulphoxide and stored in liquid nitrogen. For passaging, hMSCs were cultured for 3 days on TCPS at an initial density of 4,000 cells/cm^2^ in expansion media, detached with 0.05% trypsin‐EDTA, centrifuged, and replated at the same density. Expansion media consisted of low glucose (1 ng/mL glucose) Dulbecco's Modified Eagle Medium (ThermoFisher) supplemented with 10% FBS (ThermoFisher), 1 ng/ml fibroblast growth factor basic (Life Technologies), 50 U/ml penicillin (ThermoFisher), 50 μg/ml streptomycin (ThermoFisher), 0.5μg/ml of Amphotericin B (ThermoFisher). This method was repeated to generate desired passage numbers. For subsequent analyses, cells at desired passage numbers (P2 for early, P5–P7 for middle, and P11–P12 for late) were frozen in cell freezing medium (Sigma) and stored in liquid nitrogen.

### 2.2 Hydrogel precursors

Eight‐arm 40 kDa PEG was functionalized with norbornene as previously described.^29^, ^30^ Briefly, 5‐norbornene‐2‐carboxylic acid was coupled to eight‐arm 40 kDa PEG‐amine (Jenkem) in the presence of 1‐(bis[dimethylamino]methylene)‐1H‐1,2,3‐triazolo(4,5‐b)pyridinium3‐oxid hexafluorophosphate, *N*‐([Dimethylamino]‐1H‐1,2,3‐triazolo‐[4,5‐b]pyridin‐1‐ylmethylene)‐*N*‐methylmethanaminium‐hexafluorophosphate *N*‐oxide or HATU (Sigma) and *N*,*N*‐diisopropylethylamine (Sigma), in dimethylformamide. The reaction was performed overnight at room temperature (RT). The resulting norbornene functionalized PEG (PEG‐8NB) was precipitated with cold di ethyl ether, resuspended in deionized (DI) H_2_O, dialyzed, and lyophilized. The functionality of the PEG‐8NB (~95%) was confirmed with ^1^H‐NMR by comparing the hydrogen peak associated with norbornene double bound (~6.2 ppm) to the CH_2_ groups of PEG backbone (~3.6 ppm). Eight‐arm 20 kDa PEG‐thiol (PEG‐8SH) and CRGDS were purchased from Jenkem and Bachem, respectively.

### 2.3 Hydrogel fabrication

Hydrogels were polymerized as described previously described.^30^ Briefly, polymer precursor solution was prepared by mixing 2wt/v% 40 kDa PEG‐8NB (synthesized), 20 kDa PEG‐8SH (Jenkem), 2 mM photoinitiator lithium phenyl‐2,4,6‐trimethylbenzoylphosphinate (LAP), and 2 mM CRGDS adhesive peptide (Bachem) in PBS at a thiol:ene ratio of 1. The photoinitiator LAP has been used extensively in our group^31^, ^32^ and others^33^, ^34^ and has been shown to be cytocompatible. After vortexing, 12 or 50 μl of the solution was pipetted onto a hydrophobic Sigmacote (Sigma) treated slide. Sigmacote‐treated slides were made by flaming glass microscope slides (VWR, 3″ × 1″ × 1 mm), soaking in Sigmacote for 30 min, washing thoroughly with DI water, and air drying. A 12 mm or 25 mm thiolated coverslip was placed on top of the droplet, and it was allowed to spread fully. Glass coverslips (VWR) were thiolated by vapor deposition of (3‐mercatopropyl) triethoxy‐silane performed overnight at 80°C. The polymer precursor solution was photopolymerized in between a sigmacote‐treated glass slide and a thiolated coverslip with exposure to 365 nm UV light at 10 mW/cm^2^ for 3 min to form hydrogels with a diameter of 12 or 25 mm and thickness of 100 μm. Hydrogels were equilibrium swollen in sterile PBS overnight before use.

### 2.4 Rheological characterization

All rheological measurements were performed using a DHR3 rheometer (TA instruments) fitted with a UV light guide accessory with an 8 mm parallel plate tool. Optically thin hydrogels with a thicknesses of 250 μm were formed *in situ* by irradiating with 365 nm light (*I*_0_ = 10 mW/cm^2^, Omnicure 1,000, Lumen Dynamics) for 30 s. The shear storage modulus (*G′*) was characterized at constant strain (1%) and angular frequency (1 rad/s). The Young's modulus, *E*, was calculated using the following relationship.

*E* = 2*G*^′^(1 + ν), where a Poisson's ratio (*ν*) of 0.5 for the PEG hydrogels was assumed.^35^

### 2.5 hMSC culture on hydrogel and TCPS surfaces

Frozen down hMSCs were re‐suspended in experimental media (low glucose Dulbecco's Modified Eagle Medium [1 ng/ml glucose]; ThermoFisher) supplemented with 10% FBS (ThermoFisher), 50 U/ml penicillin (ThermoFisher), 50 μg/ml streptomycin (ThermoFisher), and 0.5μg/ml of Amphotericin B (ThermoFisher). hMSCs were then seeded on the hydrogels at a density of 4,000 cells/cm^2^ for immunostaining and 6,000 cells/cm^2^ for flow cytometry and secretome analysis. Hydrogels were moved into a new well plate 24 hr postseeding to eliminate any confounding influence of hMSCs that may have attached to the well plate during seeding. In parallel experiments, hMSCs were also seeded onto TCPS controls for flow cytometry and secretome analysis and glass coverslips for YAP and 5‐ethynyl‐2′‐deoxyuridine (EdU) analysis using the same procedure.

### 2.6 Immunostaining

hMSCs on hydrogels were fixed by treatment with 2% paraformaldehyde (PFA) for 15 min and subsequently fixed with 4% PFA for 30 min. Because of the weaker cell‐matrix interactions, hMSCs cultured on hydrogels were initially fixed with 2% paraformaldehyde for 15 min before full fixation to prevent their detachment during media aspiration. TCPS samples were only fixed for 30 min with 4% PFA. Samples were washed three times with PBS for 10 min at RT and subsequently permeabilized with 0.1% TritonX100 in PBS for 1 hr at RT. Next, samples were blocked with 5% bovine serum albumin (BSA) in PBS for 1 hr at RT. Samples were incubated with anti‐YAP antibody (1:400, mouse, Santa Cruz Biotech), anti‐CD90 (1:200, rabbit, Abcam), or anti‐CD105 (1:800, mouse, Abcam) primary antibodies in 5% BSA for 1 hr at RT or overnight at 4°C. After washing with PBST (0.5 wt% Tween‐20 in PBS) three times for 10 min, samples were incubated with secondary antibodies goat anti‐mouse AlexaFlour 647 (1:400, Invitrogen), goat anti‐rabbit Alexaflour 488 (1:400, Invitrogen), DAPI (1:500, Sigma), and Rhodamine Phallodin (1:300, Sigma Aldrich) for 1 hr in the dark at RT. Samples were rinsed with PBST three times for 10 min and stored in PBS at 4°C until imaging. YAP samples were imaged with a spinning disk confocal microscope (Operetta High Content Imaging System, Perkin Elmer). CD90 and CD105 samples were imaged using a laser scanning confocal microscope (Zeiss LSM 710).

### 2.7 Proliferation

A Click‐iT EdU Imaging Kit (ThermoFisher) was used to characterize proliferating cells at pre‐selected passage conditions (P1, P7, and P12) and the manufacturer's protocol followed. In brief, hMSCs were seeded on either hydrogels or coverslips and treated with 10 μM EdU 1 day postseeding. After 24 hr with EdU treatment in growth media, the typical cell cycle for hMSCs, samples were fixed by treatment with 2% PFA for 15 min and subsequently fixed with 4% PFA for 15 min. TCPS samples were only fixed for 30 min with 4% PFA. Samples were washed three times with PBS for 10 min at RT and twice with 5% BSA in PBS. All samples were permeabilized with 0.1% TritonX100 in PBS for 20 min at RT and washed twice with 5% BSA in PBS. Samples were incubated with the Click‐iT reaction cocktail containing an azide functionalized Alexa Flour 488 dye for 30 min in the dark at RT. Afterward, immunostaining was continued as described before.

### 2.8 YAP nuclear localization and proliferation quantification

Using the Harmony software (Perkin Elmer), DAPI and Rhodamine Phallodin channels were used to identify the nuclear and cytoplasmic region of each cell in a single imaging plane. Using the YAP fluorescent channel, the average YAP intensity in the nuclear and cytoplasmic areas was calculated for each cell. Next, the YAP nuclear to cytoplasmic ratio was calculated as the average YAP intensity in the nucleus divided by the average YAP intensity in the cytoplasm. For proliferation, the total number of nuclei was calculated using DAPI staining. Numbers of proliferating cells were quantified by the nuclei stained EdU+. Percent of proliferating cells was calculated for each field of view analyzed.

### 2.9 Immunophenotyping

Cells at early (P1), middle (P4), and late passages (P11) were seeded onto TCPS and hydrogels for 1, 3, or 9 days. A subset of the cells was analyzed for Day 0 cell surface marker expression. Flow cytometry was performed on a BD FACSCelesta. Cells were trypsinized from hydrogels or TCPS plates and washed with Cell Staining Buffer (Biolegend) with centrifugation at 3.5 x *g* for 5 min twice. As the cells were trysinized in the process, their passage numbers all increased by one. The cell pellets were resuspended in 100 μl of Cell Staining Buffer (BioLegend) and stained with antibodies anti‐CD105 conjugated with Alexa Flour 488, anti‐CD90 conjugated with Brilliant Violet 421, and anti‐CD73 conjugated with PE (BioLegend) using the manufacturer's recommended antibody volume (5 μl/10^6^ cells) by incubation for 20 min on ice in the dark. Samples were washed three times with Cell Staining Buffer, resuspended in 500 μl of Cell Staining Buffer, and the manufacturer's recommended volume of 7‐AAD viability staining solution (5 μl/10^6^ cells) was added to the samples on ice for 3 min in the dark. Size gates and voltages were set using unstained cells and fluorescence minus one controls for each passage sample. UltraComp beads (Fisher) were used as positive controls.

### 2.10 Cytokine secretion analysis

Secretory profiles were assessed for early (P1) and late (P11) passage cells on TCPS and hydrogels using a Human Cytokine Array C5 (RayBiotech) and the manufacturer's protocol was followed. Hydrogels were pooled to ensure sufficient cell numbers for cytokine detection (>200,000 cells). After 3 days in media with FBS, serum‐free media was added and cell secreted protein was allowed to accumulate for 2 days. For hydrogel samples, media was concentrated using Pierce Protein Concentrators (ThermoFisher) with a 3 kDa MWCO cutoff to ensure all cytokines were retained. After sample incubation with 1 ml of media from each condition and acellular controls for 5 hr at RT or overnight at 4°C, each array was washed with manufacturer's washing buffer for three times. Next, the membranes were incubated with a biotinylated antibody cocktail for 2 hr at RT or overnight at 4°C, washed with washing buffer three times, and incubated with HRP‐streptavidin for 2 hr at RT or overnight at 4°C. After incubation of the detection buffers, chemiluminescence was detected using a charge‐coupled device camera (ImageQuant LAS 4000 GE Healthcare). Exposure and incubation times were kept constant between each condition and its controls. A control hydrogel received the same media treatment as samples in case any components from FBS became trapped in/on the hydrogel. However, this control did not show increased cytokine detection, indicating hydrogels did not sequester any measurable amount of cytokines and only hMSC secreted factors were analyzed. Raw images were analyzed using the 2D Array feature of ImageQuant (GE Healthcare). Background signal was subtracted and average intensities were normalized to positive spot controls. Intensities from corresponding spots from control arrays were subtracted and each value was normalized to cell number.

### 2.11 Statistics

All experiments were performed with at least three replicates per condition unless stated otherwise. For proliferation, at least 20 fields of view were analyzed. For YAP analysis, at least 50 cells were analyzed per replicate, and for flow cytometry, conditions were analyzed three times and the %CD90+CD105+CD73+ cells averaged. Data were compared using one‐way ANOVA assuming unequal variances in Prism 6 (GraphPad Software, Inc). Data are presented as mean ± standard deviation.

## 3 RESULTS

### 3.1 Hydrogel characterization

**Figure 1:**
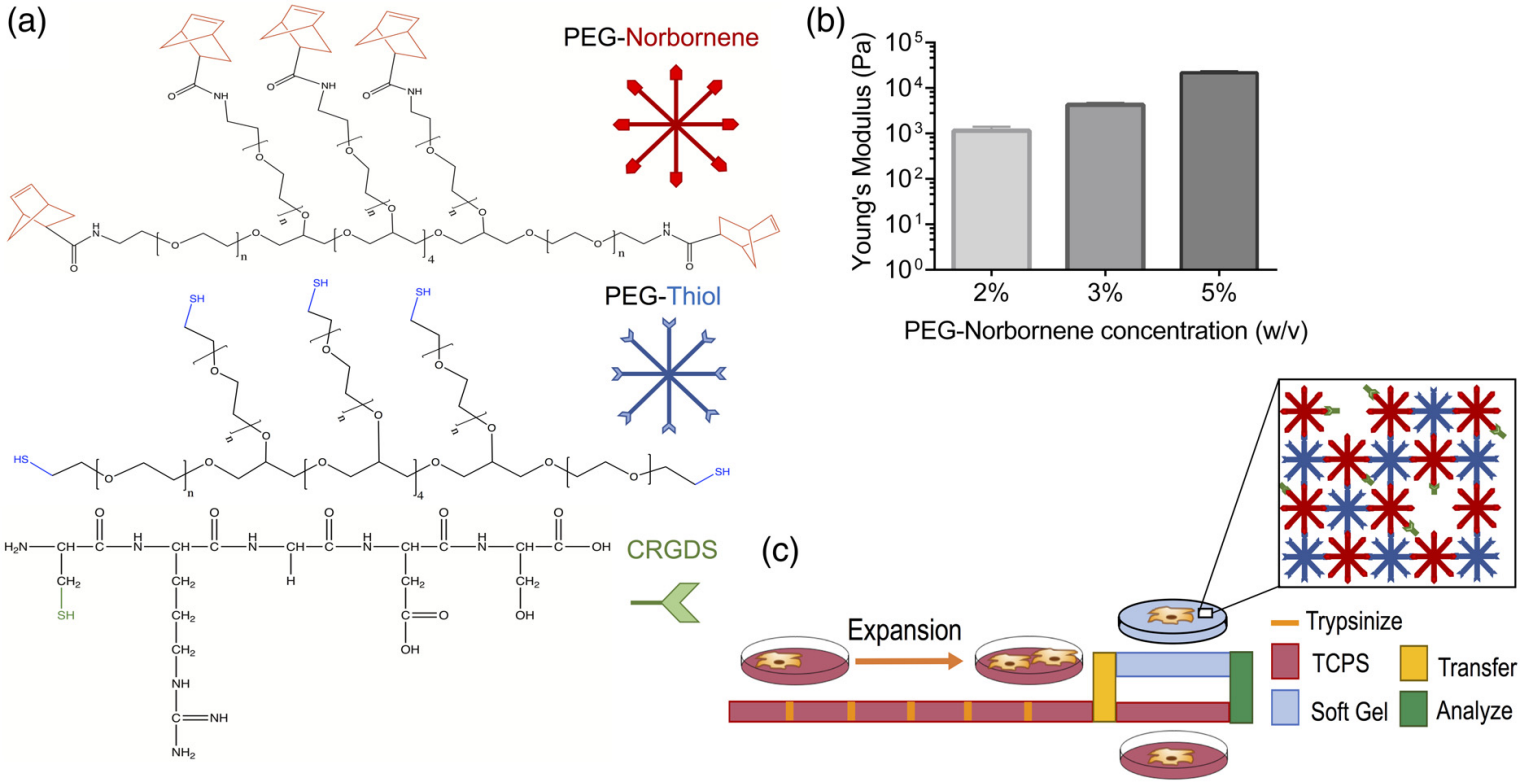
Synthesis and rheological characterization of hydrogels. (a) Structures of hydrogel precursors eight‐arm 40 kDa PEG functionalized with norbornene, eight‐arm 20 kDa PEG‐thiol, and peptide CRGDS, a fibronectin mimic. (b) Young's modulus of hydrogels of gels with 2, 3, 5 w/v% PEG‐NB and polymerized in the presence of the photoinitiator LAP. Polymer precursor solution was exposed to 365 nm light with an intensity of 10 mW/cm^2^ for 30 s. (c) hMSCs are expanded on TCPS and at preselected passage numbers transferred to PEG hydrogels. Key hMSC properties were measured at various stages of TCPS expansion and times on hydrogel materials

Peptide‐functionalized PEG hydrogels were synthesized via a thiol‐ene photoclick reaction.^29^ Eight‐arm PEG‐thiols were co‐polymerized with eight‐arm PEG‐norbornene (equal stoichiometry) to form predominantly elastic hydrogels (*E* = 1–20 kPa), where the final modulus was controlled by the concentration of PEG macromolecules in the initial solution (Figure 1a,b). The fibronectin‐derived integrin binding motif, CRGDS, was incorporated into the hydrogels at a 2 mM concentration to promote hMSC attachment.^20^ While the hydrogels biochemical and biomechanical properties can be further tuned by selection of the initial formulation, all future studies used the 2 w/v% hydrogels (*E* ~ 1 kPa). This modulus was selected as prior literature has reported the modulus of bone marrow to be ~300 Pa.^36^, ^37^ The gel formulation also provided structural integrity during fabrication, culturing and transferring of cells. Furthermore, hMSCs cultured on hydrogel substrates with this elastic modulus had largely cytoplasmic YAP and remained proliferative. Thus, we aimed to compare the differences between hMSC properties when expanded on TCPS, where YAP nuclear localization predominates and the modulus is 6 orders of magnitude higher than these PEG hydrogel microenvironments. We were particularly interested in whether or not transfer of hMSCs to the PEG hydrogels post TCPS culture would allow them to recover their initial phenotype that might drift with TPCS expansion (Figure 1c).

### 3.2 TCPS expansion leads to loss of proliferation

**Figure 2:**
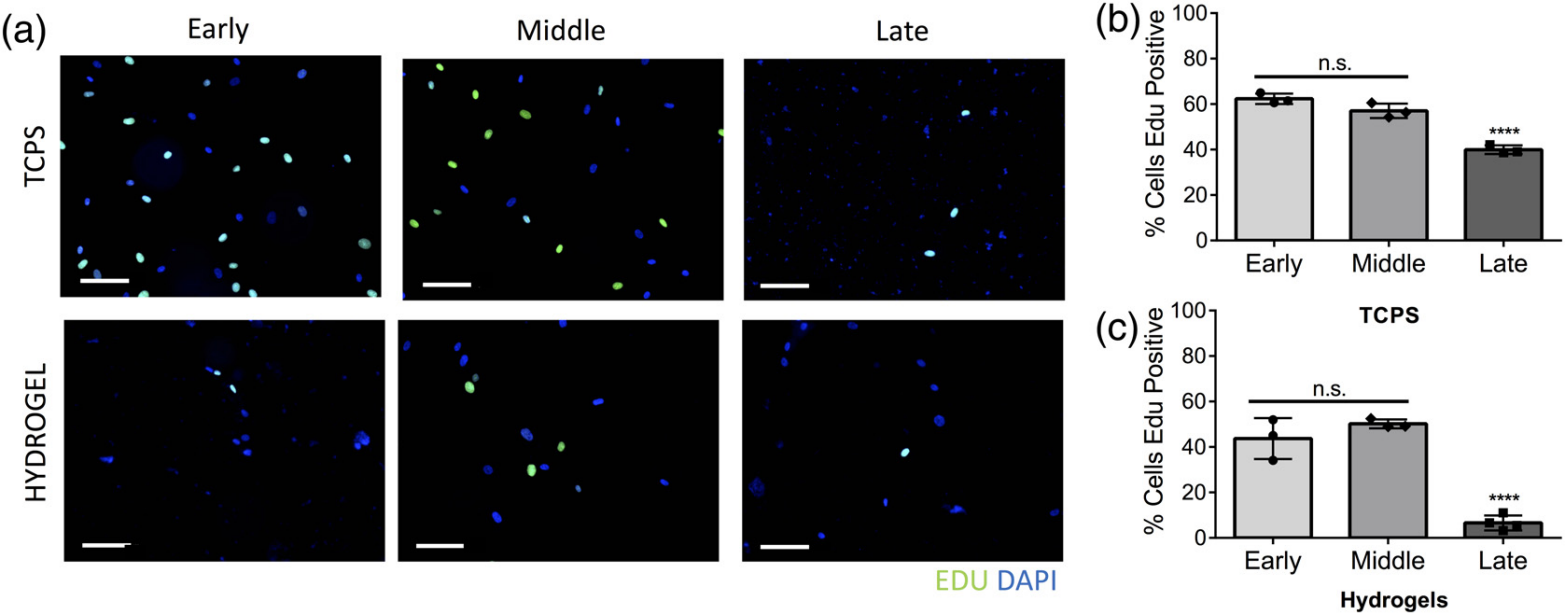
hMSCs become less proliferative with expansion. (a) Representative immunofluorescence images of early (P1), middle (P7), and late (P12) passage hMSC cell nuclei (DAPI, blue). EdU+ cells (green) represent the faction of proliferating hMSCs over a 24 hr period of culture on either TCPS or hydrogels (scale bars = 100 μm). (b) Quantification of cell proliferation shows decreased proliferation at late passages on TCPS and (c) hydrogels (n.s., not significant, ****p < 0.0001)

**Figure 3:**
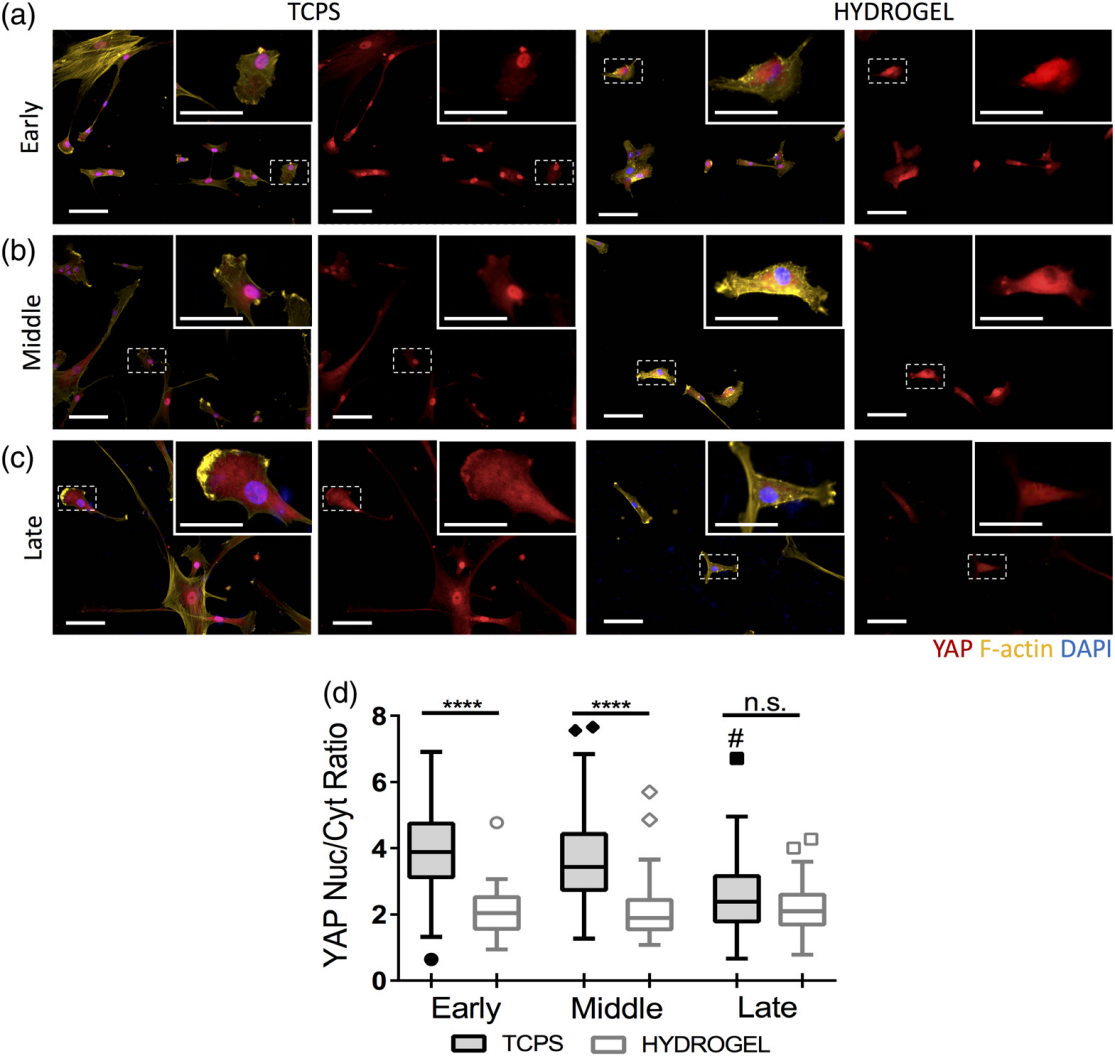
hMSC mechanosensing ability becomes lost with expansion on TCPS. Representative immunofluorescence images of (a) early (P1), (b) middle (P7), and (c) late passage (P12) hMSCs on TCPS and hydrogel conditions (scale bars = 100 μm). Insert shows a higher magnification of a single hMSC identified by the box with dashed lines (scale bar = 50 μm). (d) Tukey plot reporting the YAP nuclear to cytoplastic ratios for each cell cultured on either TCPS or the hydrogel conditions. Statistics were performed on the mean YAP nuclear to cytoplasmic ratio for each condition (*n* = 3; #‐late TCPS relative to early TCPS, ****p < 0.0001, ^#^ *p* < 0.0001, n.s., nonsignificant)

hMSC proliferation was quantified under growth conditions using an EdU assay; the effects of serial expansion on the percentage of proliferating cells are reported in Figure 2. Early (P1), middle (P7), and late (P12) passage cells were exposed to EdU for 24 hr (a typical cell cycle for hMSCs) to ensure all cells had the opportunity to proliferate. Incorporation of EdU+ was measured using immunofluorescence staining and imaging; the number of EdU+ positive cells was normalized to all nuclei to determine the percent of proliferating cells for each condition (Figure 2a). On TCPS, high levels of proliferating hMSCs were observed initially, 62 ±2% at early passages, but this level decreased with culture time and passaging (57 ± 3%, middle passages, and 39 ± 2%, late passages; Figure 2b). In contrast, hMSCs cultured on soft hydrogels had lower proliferation rates (44 ± 9% of early passages, 50 ± 2% at middle passages, and 7.0 ± 3% at late passages (Figure 3c). Overall, hMSCs show decreased proliferation with expansion and their proliferation was lower on hydrogels relative to TCPS.

### 3.3 hMSC mechanosensing ability is lost with TCPS expansion

In addition to decreased proliferative capacity, hMSC mechanosensing ability was assessed at early (P1), middle (P7), and late passages (P12). After 3 days of culture on either hydrogels or TCPS, YAP, a transcriptional co‐activator that translocates to the nucleus on stiff substrates, was fluorescently labeled. The nuclear and cytoplasmic intensities were calculated using image analysis described in the methods section (Figure 2a‐c). The YAP nuclear to cytoplasmic (nuc/cyt) ratio is plotted on a Tukey plot for each cell in the hydrogel or TCPS conditions (Figure 2d).

At early passage numbers, hMSCs cultured on TCPS exhibited high YAP nuclear localization (mean YAP nuc/cyt ratio ~4) (Figure 2a) compared to hydrogels where YAP remained diffuse in the cytoplasm (mean YAP nuc/cyt ratio ~2). These results indicate a biochemical response of the hMSCs when transferred from TCPS to the lower modulus hydrogels. For middle passage cells, the YAP nuc/cyt ratio remains high on TCPS, and the hMSCs similarly sense the difference in substrate stiffness when transferred to the hydrogels indicated by diffuse YAP (Figure 2b). In contrast, the late passage cells (P12) show cytoplasmic YAP in both TCPS and hydrogel condition (Figure 2c), which was somewhat unexpected. After image analysis and quantification, no significant difference was observed in the mean YAP nuc/cyt ratios between TCPS and hydrogels for late passage hMSCs. Compared to a difference of ~2 between TCPS and hydrogel mean ratios in early and middle passage cells, no increase in YAP nuc/cyt ratio on TCPS compared to hydrogels indicates an inability of late passage hMSCs to sense their stiff microenvironment (Figure 2d).

### 3.4 hMSC secretory properties decline with passage number on TCPS

**Figure 4:**
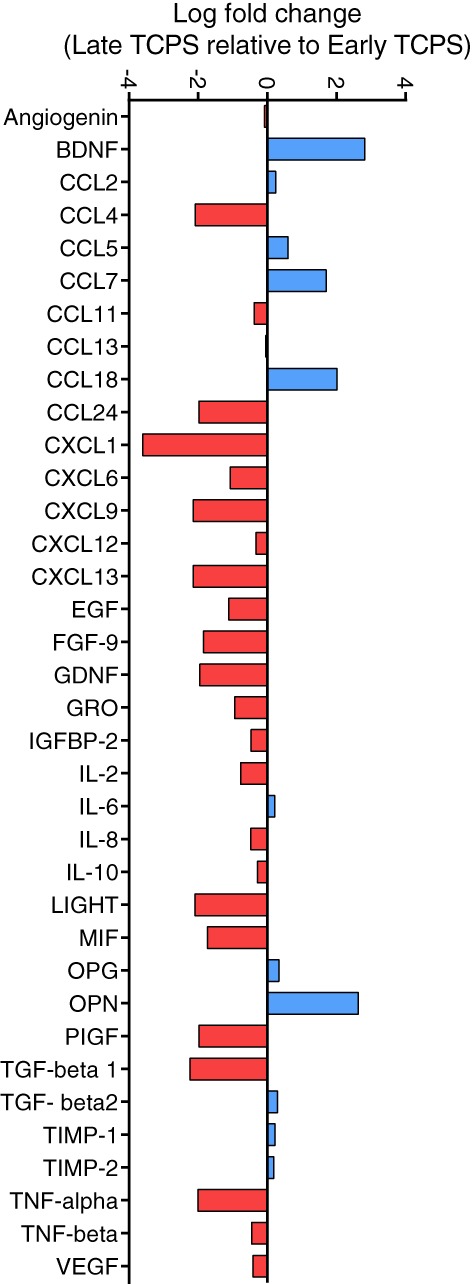
Expansion of hMSCs on TCPS decreases their cytokine secretion. Log fold change in cytokine secretion of late (P11) relative to early (P1) passage hMSCs expanded on TCPS. Cytokines with no significant changes in their secretion are not reported

hMSCs are known to secrete various cytokines and chemokines which influence the function and regenerative capacity of multiple immune cell types. Cytokine secretion from early (P1) and late (P11) passage cells was measured using a Human Cytokine Array C5 (Ray Biotech). Late passage hMSCs show decreased secretion of most cytokines and growth factors compared to early passage cells (Figure 4). Chemokine (C‐X‐C motif) ligand 1 (CXCL1) and chemokine (C‐C motif) ligand 4 (CCL4) are both involved in neutrophil trafficking and show decreased expression.^38^ Pro‐inflammatory cytokines TNF‐α and MIF were also reduced, indicating that late passage hMSCs may be less effective in promoting M1 polarization or participating in the initial stages of wound healing.^15^, ^39^ Osteopontin (OPN) was one of the few molecules whose secretion increased with passage on TCPS; OPN is known to be involved in osteogenesis and inflammatory signaling.^40^

### 3.5 Soft hydrogels rescue the hMSC immunophenotype post TCPS expansion

**Figure 5:**
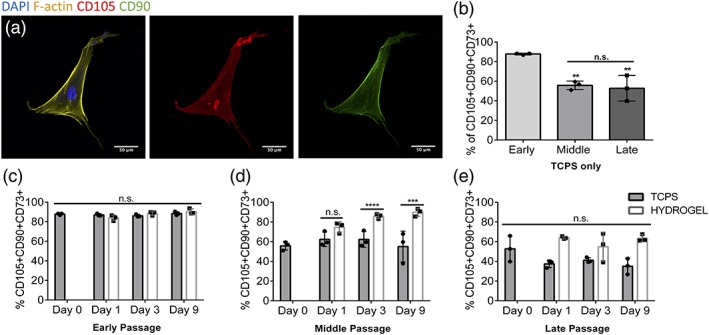
Immunophenotypic markers of hMSCs are lost with expansion on TCPS, but can be recovered by soft hydrogel culture for middle passage cells. (a) Immunofluorescence image of early passage (P2) cells on TCPS showing expression of CD105 (red), CD90 (green), F‐actin (yellow), and DAPI (blue; scale bar = 50μm). (b) Quantification of cell surface marker expression with flow cytometry of early (P2), middle (P5), and late (P12) passage hMSCs on TCPS. Quantification of cell surface marker expression for early (c), middle (d), and late (e) passage hMSCs cultured on soft hydrogels and TCPS for 1, 3, 9 days (**p < 0.01, ***p < 0.001, **** *p* < 0.0001; n.s., nonsignificant)

hMSCs' cell identity is defined by their expression of cell surface markers CD90, CD105 and CD73.^1^ The expression of these markers is also known to decrease as cells begin differentiating and lose multipotency.^41^ hMSC expression of both CD105 and CD90 was first confirmed using immunofluorescence imaging with P2 cells on TCPS (Figure 5a). The population was further characterized using flow cytometry to measure expression of three (CD105, CD90, and CD73) hMSC‐specific surface markers. Expression of these makers was quantified for hMSC populations at early (P2), middle (P5), and late passage (P12), for cells expanded on TCPS, as well as cells transferred to hydrogels. Initially, 88 ± 1% of early passage hMSCs on TCPS were CD90+CD105+CD73+ (Supporting Information Figure S1). This population of cells decreased significantly for middle (56 ± 4%) and late passages (52 ± 13%); expanded on TCPS (Figure 5b). When early passage hMSCs were transferred to hydrogels post TCPS expansion, results showed that they maintained their cell surface marker expression over the entire 9 day experimental time course (Figure 5c). Strikingly, middle passage hMSCs on TCPS (56 ± 4%) were able to recover their immunophenotype when transferred to soft gels, with 86 ± 3% of the population expressing CD90, CD105, and CD73 after just 3 days on the hydrogels (Figure 5d). This ~50% increase compared to TCPS controls was maintained over the course of 9 days on the hydrogels. In contrast, late passage hMSCs showed no recovery of their immunophenotype when transferred to the hydrogels, suggesting an irreversible change in the hMSC population after extended culture times on TCPS (Figure 5e).

### 3.6 Enhanced hMSC secretome on hydrogels

**Figure 6:**
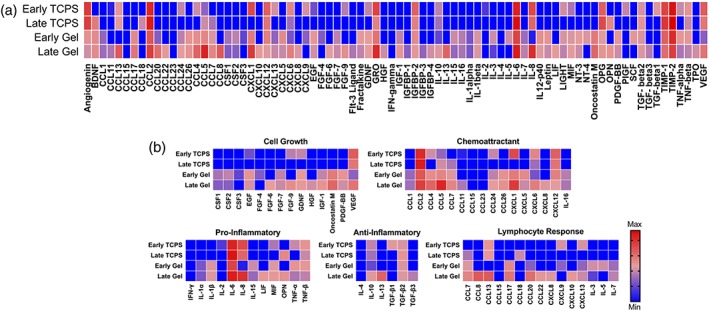
Secretory properties of hMSCs is enhanced of soft hydrogels. (a) Heatmap of cytokine secreted by hMSCs on early TCPS, late TCPS each relative to TCPS control, and early hydrogel, and late hydrogel each relative to hydrogel control. (b) Relative secretion of cytokines related to cell growth, chemoattractant, pro‐inflammatory, anti‐inflammatory, or lymphocyte response functions

Finally, the influence of transferring hMSCs to soft hydrogels on their secretory properties was measured using a cytokine array. Compared to their TCPS controls, secretion of most cytokines increased when either early or late passage hMSCs were cultured on hydrogels (Figure 6a). TCPS‐induced loss of cytokine secretion including GNDF, CXCL5, TNF‐α, and MIF was recovered on hydrogels. Secretion of cytokines involved in cell growth, including EGF, FGF‐7, IGF‐1, GNDF, and PDGF‐BB, was increased in both early and late passage cells on hydrogels (Figure 6b). Secretion of most chemoattracts, involved in macrophage and neutrophil cell trafficking, was also higher for both passages. Pro‐inflammatory cytokine secretion, known to be involved in M1 macrophage polarization and wound healing cascades, was also higher on soft gel culture. In contrast, anti‐inflammatory cytokine expression, such as those related to M2 polarization, was largely unchanged. Secretion of cytokines and chemokines involved lymphocyte responses, such as T‐cell differentiation and proliferation or B‐cell activation, were more variable and no clear trends were observed. Overall, these results indicate that soft gel culture can recover and enhance hMSC secretory properties across multiple passages.

## 4 DISCUSSION

hMSCs show promise for future cellular therapies as they are relatively easy to isolate and have been implicated in the treatment of a variety of diseases due to their differentiation capacity and secretory properties. As a cell‐based therapy, they are often administered intravenously. This requires cell numbers on the orders of tens of millions per patient per dose, necessitating expansion *ex vivo*. As hMSCs are found in multiple locations *in vivo*, their *in vivo* properties could be variable based on their specific stem cell niche. To forgo this variability, this study and various others have chosen to define the hMSC phenotype based on the *in vitro* properties of a population of freshly isolated hMSCs. Compared to early passage cells, results suggest a decrease in proliferative capacity, mechanosensing ability, cell surface marker expression, and secreted cytokines for hMSCs expanded on TCPS. However, some passages of TCPS expanded hMSCs can regain immunophenotypic markers and higher levels of cytokine secretion when transferred to soft PEG hydrogel matrices.

Previous studies have shown reduced proliferation, differentiation capacity and the onset of replicative senescence for hMSCs expanded on TCPS.^24^, ^25^, ^26^, ^27^ As these cell types are further investigated, their mechanosensing and secretory properties have become of interest for their use in tissue engineering and cellular therapies. Loss in mechanosensing with expansion could have widespread implications for the design of biomaterials and scaffolds for hMSC therapeutics. The mechanical properties of materials have already been tuned to direct stem cell fate.^19^ Decreased cytokine expression with expansion would likely lower effectiveness of therapies reliant on factors secreted by hMSCs, such as graft versus host disease. Further, only hMSC populations positive for CD90, CD105 and CD73 over a specific threshold, usually 90–95%, are currently being administered to patients.^6^, ^42^ In this work, cell surface marker expression was increased for middle passage hMSCs by transferring them to hydrogels post TCPS expansion. The percent of triple CD90+CD105+CD73+ cells increased from 56% to over 80% after 3 days on hydrogels, compared to their TCPS control (Figure 5d). Cell surface marker recovery strategies like this could be of use in hMSC manufacturing, allowing for decreased expansion times while still achieving high cell numbers.

With respect to mechanosensing, significant differences were observed between middle and late passage hMSCs. As indicated by the higher mean YAP nuc/cyt ratios (Figure 3b), middle passage hMSCs remain sensitive to the culture substrate stiffness. Thus, culture on a substrate with an elasticity similar to their *in vivo* niche may have prompted the cells to begin restoring their cell surface markers when transferred to hydrogels. In addition, about half of the middle passage cells were still able to proliferate on hydrogels, increasing turnover (Figure 2c). In contrast, the late passage hMSCs lose their responsiveness to the mechanical properties of their microenvironment, and with their low proliferation rates on hydrogels, are unable to recover their immunophenotype. However, both differences in the stiffness and biochemical surface properties of the hydrogels and TCPS are substantially different and could influence cell‐matrix interactions. The thiol‐ene PEG hydrogel system was formulated to present a single integrin‐binding RGDS epitope, while TCPS is a surface that is highly modified with adsorbed serum proteins. As a result, hMSC‐material interactions and the strength of adhesion vary between the two systems. Increased cytokine secretion on hydrogels for both early and late passage hMSCs, the first being able to sense stiffness and another unable, could indicate that the change in surface chemistry from TCPS to hydrogel is involved in promoting hMSC secretory abilities. Additionally, this increase could indicate a connection to other mechanical sensing pathways, independent of YAP, that could still be active at late passages. Overall these results indicate that both expansion time and soft gel culture have an effect on the hMSC cytokine secretion. To further increase cytokine production, preconditioning strategies with pro‐inflammatory molecules have been employed by other groups.^14^, ^43^, ^44^ As cells were not pretreated in any way, further experiments help elucidate the effect of IFN‐γ, IL‐1β, or TNF‐α simulation on secretion properties for early or late passage cells. As their immunomodulary and inflammatory response is better understood and defined, hMSC culturing conditions should be tailored to ensure maximum therapeutic potency.

Ultimately, it is important to recognize that each component of the hMSC phenotype is linked to the performance of another. For example, mechanical stiffness of microenvironment, sensed through integrins on the cell surface, can direct differentiation. Additionally, the cell surface marker CD73 has been shown to enhance immunosuppression by reducing inflammatory molecules in both B‐cells and hMSCs, useful in treating autoimmune disorders like rheumatoid arthritis.^45^, ^46^ In umbilical cord derived hMSCs, loss of CD105 expression has been linked to decreased ability to inhibit Th1 lymphocyte proliferation in co‐culture.^47^ Decreased hMSC secretory potency and chemokine receptor expression can reduce homing ability to injured tissues.^48^ The results of this study indicate that loss of properties is also linked. Loss in hMSC properties *in vitro* can have detrimental effects during *in vivo* transplantation. The success of stem cell therapies is contingent on the design of biomaterial systems to expand multipotent and regenerative hMSCs. By recovering immunophenotype and improving cytokine secretion during expansion, *in vivo* therapeutics of hMSCs could be improved.

## 5 CONCLUSION

The goal of this study was two‐fold: quantify the phenotypic drift of hMSCs during expansion on TCPS and then assess whether transfer of hMSCs to soft hydrogel matrices could restore lost phenotype. Expansion solely on TCPS decreased hMSC proliferation rates, mechanosensing ability, cell surface marker expression, and secretory profile. Transfer of middle passage hMSCs to PEG hydrogels formed via a thiol‐ene bioclick reaction (*E* ~ 1 kPa) was able to restore expression of CD90, CD105, and CD73, cell surface markers crucial to hMSC definition and function. In contrast, late passage hMSC (P12) lost their YAP‐associated mechanosensing and had low proliferation rates, and transfer to hydrogels was unable to recover their immunophenotype. In addition, culture of hMSCs on hydrogels promoted cytokine and chemokine secretion from both early and late passage hMSC populations. The simultaneous quantification of changes in multiple cell properties with exposure to TCPS and soft hydrogel culture can inform a more optimal expansion time course designed to preserve desired hMSC properties.

## CONFLICT OF INTEREST

The authors declare no conflict of interest.

## Supporting information


**Figure S1 Flow cytometry plots of hMSCS on TCPS and hydrogels**. Representative flow cytometry plots used to determine % CD90 + CD105 + CD73+ cells for early (P2), middle (P5) and late (P12) cell populations for a,b) TCPS and c,d) hydrogels. CD90 was labeled with FITC and CD105 was labeled with Brilliant Violet 421. CD73 expression (labeled with PE) was determined for cells in quadrant 2 from CD90,CD105 plots were assessed for expression. All voltages and gates were set with unstained and single fluorescent controls. Only viable cells were used for analysis. Viability was determined with 7‐AAD Viability Stain (BioLegend).
