# Supplementary material for: Rescuing mesenchymal stem cell regenerative properties on hydrogel substrates post serial expansion
Source: Bioeng Transl Med. 2018 Sep 20;4(1):51–60. doi: 10.1002/btm2.10104 (PMC6336661; doi:10.1002/btm2.10104)
Supplement: Supplementary file 1 — Figure S1 Flow cytometry plots of hMSCS on TCPS and hydrogels. Representative flow cytometry plots used to determine % CD90 + CD105 + CD73+ cells for early (P2), middle (P5) and late (P12) cell populations for a,b) TCPS and c,d) hydrogels. CD90 was labeled with FITC and CD105 was labeled with Brilliant Violet 421. CD73 expression (labeled with PE) was determined for cells in quadrant 2 from CD90,CD105 plots were assessed for expression. All voltages and gates were set with unstained and single fluorescent controls. Only viable cells were used for analysis. Viability was determined with 7‐AAD Viability Stain (BioLegend). [file BTM2-4-51-s001.docx]

**91%**

CD90

CD105

**54%**

**51%**

Early

TCPS

Middle

TCPS

Late

TCPS

CD73

Count

Early Q2

TCPS

**99.7%**

Middle Q2

TCPS

Late Q2

TCPS

**99.3%**

**100%**

**a**

**b**

CD105

CD90

**87%**

**69%**

Middle Hydrogel Day 3

Late

Hydrogel Day 3

Early Hydrogel Day 3

**90%**

CD73

Count

Early Q2

Hydrogel

Day 3

**99.9%**

**99.9%**

Middle Q2

Hydrogel

Day 3

**100%**

Late Q2

Hydrogel

Day 3

**c**

**d**
